# Building a telepalliative care strategy in nursing homes: a qualitative study with mobile palliative care teams

**DOI:** 10.1186/s12904-021-00864-6

**Published:** 2021-10-14

**Authors:** Clément Cormi, Marie Petit, Juline Auclair, Emmanuel Bagaragaza, Isabelle Colombet, Stéphane Sanchez

**Affiliations:** 1 Pôle Territorial Santé Publique et Performance des Hôpitaux Champagne Sud, Centre Hospitalier de Troyes, 101 avenue Anatole France, CS 20718, 10003 Troyes, France; 2 LIST3N/Tech-CICO, Troyes University of Technology, F-10000 Troyes, France; 3 Unité de soins palliatifs Laroque, CHU Angers, F-49000 Angers, France; 4 Service de médecine palliative et d’accompagnement, CHU Bordeaux, F-33000 Bordeaux, France; 5 Pôle Recherche et enseignement universitaire (SPES), Maison Médicale Jeanne Garnier, F-75015 Paris, France; 6 Université de Paris, APHP Centre, F-75006 Paris, France; 7 Fondation Korian pour le Bien Vieillir, F-75008 Paris, France

**Keywords:** Palliative care, Telemedicine, Remote consultation, Nursing homes, Intermediate Care Facilities, Patient Care Team, Delivery of Health Care, Integrated, Qualitative research

## Abstract

**Background:**

Despite increasing use of telemedicine in the field of palliative care, studies about the best circumstances and processes where it could replace face-to-face interaction are lacking. This study aimed to: (1) identify situations that are most amenable to the use of telemedicine for the provision of palliative care to patients in nursing homes; and (2) understand how telemedicine could best be integrated into the routine practice of mobile palliative care teams.

**Methods:**

A qualitative study based on semi-structured focus groups (*n* = 7) with professionals (*n* = 33) working in mobile palliative care teams in France.

**Results:**

Between June and July 2019, 7 mobile palliative care teams participated in one focus group each. Using thematic analysis, we found that telemedicine use in palliative care is about navigating between usual and new practices. Several influencing factors also emerged, which influence the use of telemedicine for palliative care, depending on the situation. Finally, we built a use-case model of palliative care to help mobile palliative care teams identify circumstances where telemedicine could be useful, or not.

**Conclusions:**

The potential utility of telemedicine for delivering palliative care in nursing homes largely depends on the motive for calling on the mobile palliative care team. Requests regarding symptoms may be particularly amenable to telemedicine, whereas psycho-social distress may not. Further studies are warranted to assess the impact of influencing factors on real-life palliative care practices. Telemedicine could nonetheless be a useful addition to the mobile palliative care teams’ armamentarium.

**Supplementary Information:**

The online version contains supplementary material available at 10.1186/s12904-021-00864-6.

## Background

Annually, 40 million people worldwide require palliative care, but only 14% of them actually receive it [1]. In France, 60% of deaths are reportedly eligible for palliative care, but 75% of these patients receive insufficient, or even no palliative care [2]. Mobile palliative care teams (MPCTs) are multidisciplinary teams comprising nurses, doctors and psychologists specialised in palliative care, who are available to attend to palliative care cases within the hospital, or outside the hospital. Within the hospital, MPCTs provide support and advice for medical teams, and are generally called on to address complex situations [3]. Out-of-hospital MPCTs provide palliative care follow-up for patients in their usual place of residence (at home or in a nursing home), but these resources are limited, and widely disparate across regions. For example, the French region of Burgundy has 3 million inhabitants, and had 29 MPCTs in 2016, whereas the greater Paris area had four times more inhabitants, but only 70 MPCTs [4, 5]. These disparities have led to a rising trend in the number of hospital admissions within the last few days before death. Pennec et al. reported that two-thirds of individuals living at home or in a nursing home 28 days before death actually die in hospital [6]. Bolstering the availability of out-of-hospital palliative care services could help to limit these hospital admissions.

Due to a scarcity of medical professionals trained in palliative care [7], telemedicine has been garnering increasing popularity in recent years. Telemedicine is an approach that involves the use of information and communication technologies (ICT) to deliver remote medical care. There are two forms of telemedicine, namely synchronous and asynchronous. In synchronous telemedicine, there is a live, two-way exchange between the requester and the medical expert, whereas in asynchronous telemedicine, the data is collected and transmitted to a healthcare provider for later (offline) analysis. Telemedicine is an umbrella term that covers various situations [8, 9], such as teleconsultation (clinician to patient); tele-expertise (clinician to clinician); telemonitoring (remote collection of data for simultaneous or later interpretation), or teleassistance (a healthcare professional remotely advises another when carrying out a procedure). A major challenge is to identify the circumstances and processes where physical inter-human relationships can successfully be replaced by audio and video contacts while providing the same, or even enhanced quality of care and services.

The use of telemedicine in the field of palliative care is increasing [10, 11], associated with an improvement in symptom management [12] and a decrease in hospital admissions and emergency department visits [13]. However, the use of telemedicine in palliative care is still controversial, reflecting concerns about the erosion of the clinician-patient relationship [14]. A study by Donelan et al. summarizes existing controversies surrounding the use of telemedicine for palliative care [15]. While 60% of the surveyed physicians see no difference between an office visit and a virtual visit as regards the overall quality of the visit, about 46% of them feel a better “personal connection” with the patient during the office visit compared to a virtual visit.

Assessing the range of tele-palliative care services remains challenging because of the complexity of palliative care situations, which involve an intricate combination of clinical, social, cultural, ethical and psychological parameters [16]. In this regard, qualitative research methods hold promise as a means to assess telemedicine services [17]. With many nursing homes situated in rural or peri-urban areas, often at a distance from medical resources, or with limited medicalisation on site [18], French health authorities have invested in telemedicine equipment for many nursing homes in an attempt to improve healthcare delivery to residents [19].

In this context, we first sought to identify, from the perspective of the mobile palliative care team, which situations could be amenable to telemedicine for palliative care delivery to nursing home residents (considering all types of telemedicine activities); and second, to understand how telemedicine could be best integrated into the routine practices of mobile palliative care teams. Answering these questions could make it possible to expand the scope of telemedicine for the delivery of quality palliative care in nursing homes.

## Methods

### Design and setting

We performed a qualitative study using semi-structured focus groups with professionals working in mobile palliative care teams (MPCTs). This approach was chosen to enable free and spontaneous exchange, and to create a group dynamic [20], while also enabling expression of the multidisciplinary nature that characterises MPCTs. Furthermore, this approach is particularly useful for exploring fields where knowledge is sparse [20, 21]. The research team included two palliative care physicians, three palliative care researchers, and a computer science researcher working in the field of computer-assisted cooperation and telemedicine.

### Study population

Using the national directory of all palliative care establishments affiliated with the French Society for Supportive and Palliative Care (Société Française d’Accompagnement et de Soins Palliatifs), we defined the following inclusion criteria:

#### Inclusion criteria

- MPCT with out-of-hospital activity oriented towards nursing homes;
- MPCT comprising at least one physician specialised in palliative care, and one nurse, in line with French legislation regarding the composition of these teams.

#### Exclusion criteria

- MPCT with links to any of the researchers involved in the project;
- MPCT not available during the study period.

No other additional criteria were stipulated regarding the composition or organisation of the MPCTs, because of the wide heterogeneity across teams.

We decided to include MPCTs until data saturation was reached (i.e. the point beyond which further interviews or group discussions yield no new material) [21]. In view of the diversity of healthcare establishments and modes of operation of the MPCTs across France, we decided to contact 10 different teams meeting the inclusion criteria. A second round of selection was initially planned in case saturation was not reached with the participating teams, but was not required.

These 10 teams were contacted by mailing an information leaflet about the study to the contact email address of the healthcare establishment with which the MCPT was affiliated. Among the 10 teams contacted, seven (70%) were included. The three remaining teams never answered the request (*n* = 2) or were not available during the study period (*n* = 1). Each member of each of the participating teams was individually invited to participate in the focus groups, regardless of their profession, totaling 36 invited professionals, of whom 33 (92%) agreed to participate. The three professionals who refused did so because they were due to be on vacation on the date when the focus group meetings were being held. Participants did not receive any incentive for their participation.

### Data collection

One focus group meeting was held with each MPCT. Each meeting was facilitated by the first author (CC (male)). Each meeting started with a brief presentation of the different forms of telemedicine and followed the interview guide (See supplementary file). Using the motives for which nursing homes call on the MPCT as a starting point, participants were invited to discuss how useful telemedicine procedures could be to meet these different needs, how the different forms of telemedicine could be integrated into their routine practice, and finally, which types of management they would not envisage by telemedicine.

This format was piloted on the first participating team and was found to require no modification and thus, data from the first team were included in the analysis. With the participants’ consent, meetings were recorded and transcribed for later analysis. During the focus groups, CC also took notes to enable participant identification upon transcription.

### Data analysis

Transcripts were analysed by three co-authors (CC (male), MP and JA (female)). The first round of analysis was performed individually by each of the three co-authors the week after each meeting. The second round of analysis was performed conjointly by the same co-authors after all the material had been collected. The second round resolved differences in interpretation and built the use-case model.

The use-case model is a model of how the various users interact within the healthcare system to provide a service. This approach is widely used for software engineering to capture users’ requirements during the design process [22, 23]. In the use-case model developed here, we describe the goals of the users, as reported by our study participants, the interactions between these users and the system, and the type and suitability of telemedicine solutions in meeting the goals. This model is based solely on the experiences and examples reported by our focus group participants.

Data were analysed using thematic analysis [24, 25]. The aim of thematic analysis is to identify and categorize the different themes occurring in a cross-sectional manner across all interviews. Each theme is then considered as a meaningful and independent unit of discourse. Major themes and secondary themes are identified. Major themes are relevant points that are spontaneously well developed by all participants. Minor themes are less well developed by participants, of lesser importance in their discourse, and not necessarily mentioned by all participants.

We established that saturation was reached after five focus group meetings. All focus groups were held, recorded, transcribed and analysed in French. Key citations were translated for the purposes of publication, to illustrate the results, by a native-English speaking qualitative researcher (FE (female)).

## Results

From 17th June to 25th July 2019, seven focus group meetings were held (i.e. one with each participating MPCT), lasting on average 45 min (range 36 to 52 min). The meetings were held on the premises of each MPCT. The composition of the focus groups is detailed in Table 1. There were three teams from the North of France, one from the greater Paris area, one from the West and two from the East of France. None of the participating MPCTs routinely used telemedicine solutions prior to participating in our study.

**Table 1:** Composition of the 7 participating mobile palliative care teams

Team	Participants					
	Physician	Nurse	Psychologist	Social worker	Medical Student	Total
MPCT 1	1	2	1	0	0	4
MPCT 2	1	2	1	0	0	4
MPCT 3	1	3	1	Absent for holidays	1	6
MPCT 4	2	1	Absent for holidays	0	0	3
MPCT 5	2	3	1	1	0	7
MPCT 6	2	2	1	0	0	5
MPCT 7	2	2	Absent for holidays	0	0	4
Total	11	15	5	1	1	33

### Tele-palliative care: navigating between usual and new practices

In this section, all forms of telemedicine activities were considered, i.e. tele-consultation, tele-expertise, tele-assistance, tele-monitoring and remote medical triage. The focus group participants gave their opinions about whether and how these activities could be integrated into their usual practice, during their usual interactions with nursing homes.

Although none of the participating teams routinely used telemedicine as defined by law, they all considered that tele-expertise is already an integral part of daily practice for MPCTs, since they regularly provide synchronous advice to healthcare providers in nursing homes over the telephone.

*“I’d say that we already do “tele-expertise” because we give advice over the phone. So I think it’s quite relevant.” (MPCT 3)*

The participants felt that the use of teleconsultations (i.e., synchronous clinician-to-patient video consultation) could be envisaged under certain conditions. The objective of the tele-consultation would have to be very clearly defined at the time it was being planned (e.g., need to adjust an analgesic medication), and the focus group participants felt that a healthcare professional (physician or nurse) would have to be present at the patient’s bedside. This choice of physician or nurse would have to be made according to the pre-defined objective of the teleconsultation. The chosen healthcare professional would in any case have to be a staff member of the nursing home, because the purpose of MPCTs is to give advice and support, not to take over patient management. The MPCTs provide guidance, but nursing home staff continue to deliver the care.

*“Teleconsultations could be useful, especially if there’s a physician on the other end, or a nurse for wounds. It’d be interesting to be able to see what the patients look like, to get an idea of how uncomfortable they are. You don’t get that over the phone.” (MPCT 2)*

The participants also expressed the sentiment that telemedicine could enable new practices. While they felt that tele-monitoring of vital signs in real-time is of limited practical use for MPCTs, obtaining the data from connected patient-controlled analgesia pumps (PCA) containing morphine would make it possible to monitor the patient’s pain and guide nursing home teams in adapting analgesia as required. Although equipment for monitoring this type of data is commercially available, our participants reported that it is not widely used in their practice. The participants further stated that teleassistance could provide support to caregiving teams on-site during the performance of painful nursing care procedures, by guiding the caregivers’ movements, for example during bed-bathing or oral care.

### The tele-palliative care project environment: “influencing factors”

Based on situations described by the focus group participants, we identified circumstances likely to mediate the probability of the MPCT choosing to use telemedicine to address that situation. We describe below the factors that positively (make it more likely) or negatively (make it less likely) influence the choice to use telemedicine, as reported by the focus group participants.

#### Circumstances that facilitate the use of telemedicine

The focus group participants reported that in order to use telemedicine solutions more widely, they need to have a good degree of autonomy regarding when and how to implement it, and a high level of flexibility regarding the practical organisation, which would be dependent on the requesting nursing homes.

*“[We’d] have to maintain a certain level of room for manoeuver, like that we could choose ourselves which situation would be suitable. […] I suppose that for it to work, there’d already need to be a good level of communication, and a climate of trust [with the nursing homes].” (MPCT 6)*

Secondly, the participants expressed a need for all those involved to be adequately trained in the implementation of telemedicine activities.

*“On the ground, the partners we work with would have to be trained, and we would too.” (MPCT 6)*

#### Obstacles to the use of telemedicine

Conversely, the participants cited certain medical or organisational constraints that, in their opinion, would make them reluctant to use telemedicine.

In medical terms, the participants cited in particular the dying moments of a patient, and unanimously stated that they could not envisage telemedicine in this situation.

*“I couldn’t look at someone agonising on the screen and give them advice.” (MPCT 3)*

In terms of organisation, the participants expressed a fear that telemedicine might be used by the government or health authorities as a pretext to re-organize healthcare delivery or even as a pretext to introduce cutbacks.

*“My second [fear] would be that the aim would no longer be to improve management, but rather to cut back on resources […], especially human resources, whereas that’s fundamental for the quality of care and accompaniment.” (MPCT 4)*

In this regard, the participants underlined the importance of not losing sight of the main objective, which is ultimately to help the patient.

*“There’s a sort of general excitement about these tools, but the aim is not to do telemedicine, the aim is to have the right tools to practice good medicine.” (MPCT 4)*

### A use-case model of telepalliative care

**Fig. 1:**
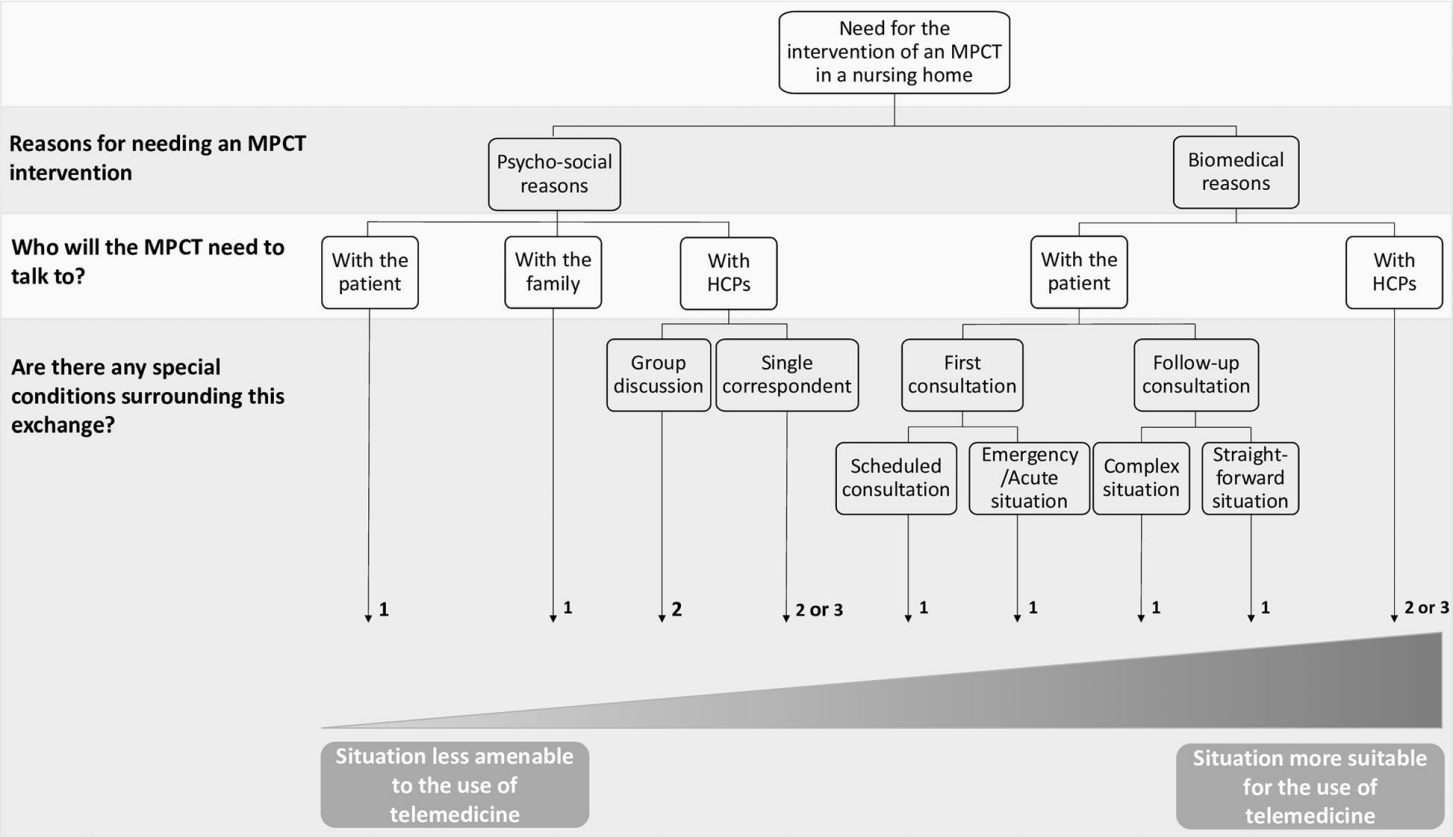
A use-case model of telepalliative care. HCP: Healthcare provider; 1: Teleconsultation; 2: Synchronous tele-expertise; 3: Asynchronous tele-expertise

The conceptual framework to emerge from the analysis of the focus groups is presented in Fig. 1. It shows a gradient of situations that MPCTs may encounter, as reported in our focus groups, according to whether or not the situation could be suitable for the use of teleconsultation and/or tele-expertise. The gradient extends from the situations least amenable to management with teleconsultation and/or tele-expertise (left-hand side) towards the most favourable situations (right-hand side), from the perspective of the teams participating in the present study. We did not include tele-assistance and tele-monitoring in the Figure, since the focus group participants did not expressly cite these forms of telemedicine as solutions that they would use regularly. In their projections about potential wider use of telemedicine, the most attractive options that were cited most often by the participants were teleconsultation and tele-expertise.

The motive for calling on the mobile palliative care team influences the likelihood that telemedicine could be used to deal with the request. Indeed, management of physical symptoms (pain, dyspnea, etc.) seems to be more amenable to telemedicine. Requests for psychological support, ethical dilemmas or management of conflict tend to necessitate a face-to-face meeting and are thus less amenable to management using telemedicine.

*“There’s a part of our work that couldn’t be done [by telemedicine]… I’m thinking of support for families… it’s just not possible.” (MPCT 6)*

In the focus groups that included a psychologist (all except MPCT 4 and MPCT 7), the psychologists were unanimous in declaring that they simply could not use telemedicine.

*“The rationale is just not the same… I can’t see myself doing psychological follow-up [by telemedicine].” (MPCT 6)*

When the request from the nursing home is unclear, telemedicine could be useful to identify the patient’s needs. The choice between tele-expertise (from professional to professional) and teleconsultation (between physician and patient) would depend on whether there is a need to see the patient, but in any case, the telemedicine procedure could serve as a screening tool to guide management.

*“In situations where the request is not very clear, I think it could be helpful to do a teleconsultation first, and then see if the request is justified, before moving on to a physical meeting.” (MPCT 5)*

The teams participating in our study see telemedicine as being more suited for the follow-up of inpatients leaving palliative care units they have already seen in person. A teleconsultation could always be performed between two in-person visits, or to prepare a future in-person consultation.

*“I think it would be good for follow-up situations, for certain specific procedures.” (MPCT 5)*

*“It would be a complement to on-site visits in most situations.” (MPCT 3)*

In acute situations (e.g., a patient whose status deteriorates rapidly and seriously, on a Friday evening at 5 pm), the main advantage of telemedicine would be to respond rapidly to requests, especially those from nursing homes that are geographically distant from the MPCT, thus reducing the response times, and avoiding unnecessary transfers to hospital via the emergency services.

*“Sometimes we have to defer visits because we can’t answer that day, and that means time lost for the patient […]. For sure, reducing the time delay would one of the main objectives.” (MPCT 7)*

## Discussion

This study aimed to identify situations that are most amenable to the use of telemedicine for the provision of palliative care to patients in nursing homes from the perspective of MPCTs. We further sought to understand how telemedicine could best be integrated into the routine practices of MPCTs.

According to the focus group participants, telemedicine is not used to the same intents and purposes as presence-based consultations and does not answer the same needs. Indeed, our focus group participants felt that telemedicine cannot replace face-to-face consultations. In the literature, palliative care is classically described to cover two main dimensions [26]. The first is biomedical, oriented towards symptoms, and is predominantly technical. It requires clinical skills, pain control, and use of various treatments. It would appear that this type of technical management is amenable to the use of telemedicine [27, 28], which is in line with our results. The second dimension, more psycho-social, mobilizes human relations and interpersonal skills [26, 29]. It is more difficult to envisage this type of interaction taking place without a physical meeting with the patient [27, 30]. Here, our results and graphical representation are also in line with the literature, but bring to light certain nuances from the practical experience reported by our participants, notably the “influencing factors” impacting telemedicine use. While the use of telemedicine can be envisaged during palliative care, it would appear that teleconsultations should alternate with presence-based consultations [31].

The psychologists who participated in our focus groups declared unequivocally that they did not want to use telemedicine for their patient interviews. Beyond the dialogue, consultation with a psychologist involves non-verbal communication that the psychologists are afraid would be lost through telemedicine. Yet, a sociological study investigating teleconsultations for mental health found that the distance and the screen could sometimes represent a form of protection for the patient [32]. By video-conference, the patient might feel at greater liberty to address painful subjects. This is the paradox of the distance [32]. It would be interesting to assess whether this practice could be implemented in palliative care, either with the patient or the family.

Although telemedicine could certainly be implemented without the presence of a healthcare professional at the patient’s bedside, the participants in this study felt that it was necessary for a physician or nurse to be with the patient during the teleconsultation. They cited three main reasons for this: first, many elderly patients in nursing homes might have technical difficulties operating the telemedicine programme on the computer or other support. Second, for the purpose of supportive care, it is necessary for someone to be physically there with the patient. Third, the MPCT professional provides guidance, but the nurse from the nursing home provides the care and involving them in the teleconsultation keeps them involved and up-to-date. In our opinion, a nurse from the nursing home would be the most suitable person for this job. Previous studies have shown that telemedicine changes the manner in which healthcare professionals interact and cooperate, and it leads to new patterns of task delegation [33–35]. Ordinarily, when there is a change in role distribution between professionals, it is the socially undervalued, and unpleasant work that gets delegated [36]. During a teleconsultation, by performing part of the clinical examination that is indispensable to the physician, the nurse would be performing essential tasks that are indispensable for the success of the teleconsultation [35, 37]. Telemedicine therefore requires a transfer of knowledge and responsibilities from the physician to the nurse, and could help to build capacity [38]. In this way, it contributes to role re-distribution [39], a pre-requisite for its routine use [38]. The use of telemedicine also seems to promote best practice [39]. However, further research is needed to evaluate the impact of telemedicine on enhancing skills among nursing home staff.

Furthermore, there is considerable heterogeneity in the structure and *modi operandi* of MPCTs, and in palliative care, every clinical and personal situation is unique. It has been reported that healthcare professionals themselves are often the leading cause of telemedicine project failure [14]. Our results indicate that for palliative care professionals, the tools at their disposal, and how these tools are used, must be flexible. There can be no “one-size-fits-all” model. Each project would need to be co-constructed with the professionals, patients, and families involved, with a consensual aim to serve the quality of, and greater access to care. Identifying a project manager in each healthcare establishment would be key to maximizing the chance of success [40]. In parallel, further studies could examine the specific contribution of the “influencing factors” identified in the present analysis, and the extent to which they impact on the potential for implementation of telemedicine.

Although professionals usually prefer in-person encounters with patients [41], the ongoing Covid-19 pandemic has required physicians to adapt their practices, and telemedicine has rapidly become a key part of their armamentarium [42, 43]. The need for a clinical examination is a major limitation on the use of telemedicine, but Lally et al. nevertheless reported that rapport building, and communication around serious illness translate well to telehealth. Accordingly, pain management via telemedicine solutions does not appear to pose any particular difficulty [44]. In the current pandemic context, Hawkins et al. showed that virtual visits provided a more reactive response, and enabled more regular follow-up, while at the same time protecting healthcare personnel against risk of exposure, and also saving previous personal protective equipment [45]. However, although telemedicine is a safe and effective solution put in place rapidly in response to the COVID-19 crisis, Hawkins et al. underline that its long-term use warrants further evaluation in the palliative care setting [45]. Emerging evidence from the COVID-19 pandemic and the use of telemedicine in palliative care is congruent with our results. Our findings further contribute to understanding the challenges of applying telemedicine to a diverse range of palliative care situations.

### Study limitations

The local organisation of palliative care delivery, the cultural context, representations about end-of-life, death and technology could hinder the translation of our results to other contexts [46]. To substantiate the models developed here, it would be useful to test them in real-life experiences of telepalliative care. Moreover, our design study precludes evaluation of the impact of each influencing factor identified on the use (or not) of telemedicine. Secondly, although the meetings were recorded and transcribed, non-verbal information may have been lost. Thirdly, the focus groups were performed with and analysed from the point of view of the MPCTs. Input from the nursing home professionals, and patients would be additionally informative.

## Conclusion

This qualitative study shows that depending on the motive for which the nursing home calls on the MPCT, telemedicine may be more or less suitable as a solution for the delivery of palliative care. Findings from our focus groups show that requests regarding patient symptoms may be particularly amenable to telemedicine. Conversely, according to our focus group participants, psycho-social distress in a patient likely requires presence-based consultation. Our study also identified “influencing factors” that impact on whether or not telemedicine could be used in specific palliative care situations. Further studies are warranted to study the impact of these factors on real-life practice. Real-life experience and opinions such as those reported here are key to guiding future practice in telepalliative care delivery, taking account of the needs of the palliative care professionals themselves.

## Supplementary Information

**Additional file 1: Supplementary file.** Interview guide for focus groups with mobile palliative care teams.

## Data Availability

All data generated and/or analyzed during the current study are available from the corresponding author on reasonable request.

## Abbreviations

MPCT: Mobile palliative care teams
ICT: Information and communication technologies
