# Supplementary material for: Building a telepalliative care strategy in nursing homes: a qualitative study with mobile palliative care teams
Source: BMC Palliat Care. 2021 Oct 14;20:156. doi: 10.1186/s12904-021-00864-6 (PMC8514278; doi:10.1186/s12904-021-00864-6)
Supplement: Supplementary file 1 — Additional file 1: Supplementary file. Interview guide for focus groups with mobile palliative care teams. [file 12904_2021_864_MOESM1_ESM.docx]

**Supplementary file: Interview guide for focus groups with mobile palliative care teams**

- What are the motives that prompt nursing homes to contact you?
- Do you think that certain telemedicine procedures could be useful for your practice in nursing homes? If so, which ones?
- How would you integrate them into your practice in nursing homes?
- Which parts of the work of mobile palliative care teams could NOT be performed by telemedicine?
- If your team was selected to test a telemedicine project, would you welcome the idea?
- Would you be willing to integrated telemedicine into your daily practice?
